# Research Priorities for Children With Food Allergy: A UK James Lind Alliance Priority Setting Partnership

**DOI:** 10.1111/cea.70377

**Published:** 2026-07-05

**Authors:** Roxanne M. Parslow, Farhad Shokraneh, Sara J. Brown, Mary Feeney, Graham Roberts, Hora Soltani, Joanne Walsh, Amy Dobson, Emma Vaughan, Deborah Marriage, Suzi Holland, Mary Kelly, Holly Shaw, Alicia Kennedy, Eden Byrne, Liliana Jackson, Gail Jackson, Lauren Gluyas, Jonathan Gower, Alyson Huntley, Matthew J. Ridd

**Affiliations:** ^1^ University of Bristol Bristol UK; ^2^ University of Edinburgh Edinburgh Scotland; ^3^ University of Southampton, David Hide Asthma and Allergy Centre, NIHR Southampton BRC, University Hospital Southampton NHS Foundation Trust Southampton UK; ^4^ Sheffield Hallam University Sheffield UK; ^5^ Castle Partnership Norwich UK; ^6^ University of Southampton Southampton UK; ^7^ Allcare Pharmacy Dublin Ireland; ^8^ University Hospitals Bristol, NHS Foundation Trust Bristol UK; ^9^ Eczema Outreach Support Linlithgow Scotland; ^10^ Anaphylaxis Farnborough UK; ^11^ Allergy Frimley UK; ^12^ Natasha Allergy Research Foundation London UK; ^13^ PSP steering group public member University of Bristol Bristol UK; ^14^ James Lind Alliance Southampton UK

**Keywords:** adolescents, children, food allergy, paediatrics, research priorities, young people

## Abstract

**Introduction:**

Using the James Lind Alliance (JLA) methodology, we established a Priority Setting Partnership (PSP) to identify the most important unanswered research questions in childhood food allergy. This approach places those directly affected, those with food allergy, their parents/carers, and healthcare professionals at the centre of the process.

**Methods:**

A multidisciplinary steering group (*n* = 19 people) oversaw the PSP. Research uncertainties were collected through a UK‐wide online survey distributed to children, young people and adults with food allergy, their parents/carers, and healthcare professionals working in food allergy. A focus group was conducted with seven children aged 8–11 years with food allergy to ensure inclusion of their perspectives. Submitted questions were reviewed, combined into summary questions, and checked against existing evidence to confirm that they represented genuine uncertainties. An interim prioritisation survey was used to rank questions, with equal weighting given to each stakeholder group. A final facilitated workshop used a nominal group technique to agree on the top 10 research priorities.

**Results:**

In total, 916 respondents submitted 2563 questions. After removing out‐of‐scope and already answered questions, an interim prioritisation survey was completed by 1087 participants. The final workshop involved 29 participants, including young people (*n* = 3), young adults (*n* = 4), parents (*n* = 7) and multidisciplinary healthcare professionals (*n* = 15), who agreed on the top 10 questions. These cover prevention, early diagnosis, treatment, causes, eating out, safety in care settings, impact, emergency treatment, and awareness of food allergy. There was strong consensus from the final workshop across all attendees that prevention should be the number one priority.

**Conclusion:**

Using a rigorous, transparent, and person‐centred approach, we have identified the most important research priorities in childhood food allergy. They highlight the depth and breadth of research required to improve the prevention, diagnosis, treatment, and broader impacts of food allergy on children, families, and carers who live with this condition.

## Key Messages

1. Childhood food allergy research priorities include prevention, early diagnosis, treatment, support and lived experience.
2. Patients, carers, and healthcare professionals share common priorities when setting the future research agenda
3. These coproduced research priorities provide a roadmap for funders, researchers, and policymakers.

## Graphical abstract

The UK childhood food allergy James Lind Alliance Priority Setting Partnership collaboratively identified the most important research priorities highlighting the breadth of research required to improve the prevention, diagnosis, treatment and broader impacts of food allergy on those directly impacted. These provide a clear, stakeholder‐driven agenda to guide future research and funding.

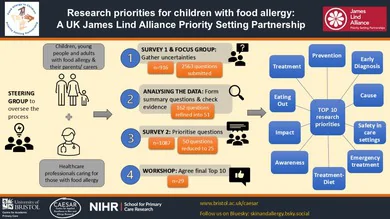

**Abbreviations**
JLA: James Lind Alliance
PSP: Priority Setting Partnership

## 1 Introduction

Childhood food allergy represents a significant health concern, affecting approximately 2%–7% of children in the United Kingdom [1, 2, 3, 4]. Hospital admission and diagnoses related to food allergy have increased, although there is uncertainty about how much of this is due to changing disease incidence versus behaviour change [5]. For example, cow's milk allergy affects less than 1% of children in Europe yet rates of milk allergy diagnosis and treatment far exceed this [6]. Children with food allergy have been found to have higher levels of anxiety, depression, and reduced health related quality of life and psychosocial functioning whilst caregivers face anxiety, financial strain, limitations in social activities and increased work absences [7, 8, 9]. Despite the high prevalence and impact of food allergy, unanswered questions remain across prevention, diagnosis, management, and lived experience.

Historically, research agendas in food allergy have been shaped predominantly by academic and commercial priorities, with limited direct input from those who live with and manage the condition daily. This disconnect means that research may not align with the needs and concerns of those living with the condition, their family and caregivers, and front‐line clinicians. The James Lind Alliance (JLA) Priority Setting Partnership (PSP) methodology offers a structured and transparent approach to redressing this imbalance by bringing together patients, carers, and healthcare professionals to collaboratively identify and prioritise unanswered research questions. Established in 2004 to ensure that research funding reflects the questions that matter most to those directly impacted, the JLA framework has been applied across a range of health conditions internationally, yielding top‐ten lists of research uncertainties that reflect shared priorities among stakeholders [10, 11, 12, 13]. PSPs identify and prioritise unanswered questions for research, or evidence uncertainties, providing a platform for researchers and funders to prioritise the research that matters most to those impacted. To date, there has been a lack of formal prioritisation work in food allergy. The UK Food Standards Agency (FSA) conducted a priority‐setting exercise (with adapted James Lind Alliance methodology) to identify key research needs for protecting consumers with food hypersensitivity. The project highlighted crucial needs for improved food allergen labelling, better management of cross‐contamination by food businesses and improved consumer education on managing risks, especially when dining out. However, the FSA exercise excluded areas related to clinical care such as diagnosis and treatment, and focused on the food system and consumer protection [14].

Following the JLA methodology, we conducted a UK‐wide priority setting process for food allergy in children to identify and prioritise research questions. This paper describes the process, the resulting top 10 research priorities and discusses the implications for future research in this area.

## 2 Methods

### 2.1 Study Design

The food allergy in children JLA Priority Setting Partnership was launched in 2024, conducted in accordance with the JLA Guidebook [15] and overseen by an independent JLA Advisor (JG) to ensure transparency, balance, and methodological rigour. The PSP engaged a broad group of stakeholders (children and young people with food allergies, their parents and carers, adults diagnosed with food allergy in childhood, healthcare professionals and allergy and eczema charities) through a steering group, systematic online surveys, a children's focus group and a final all‐day in‐person consensus workshop (Figure 1).

**FIGURE 1:**
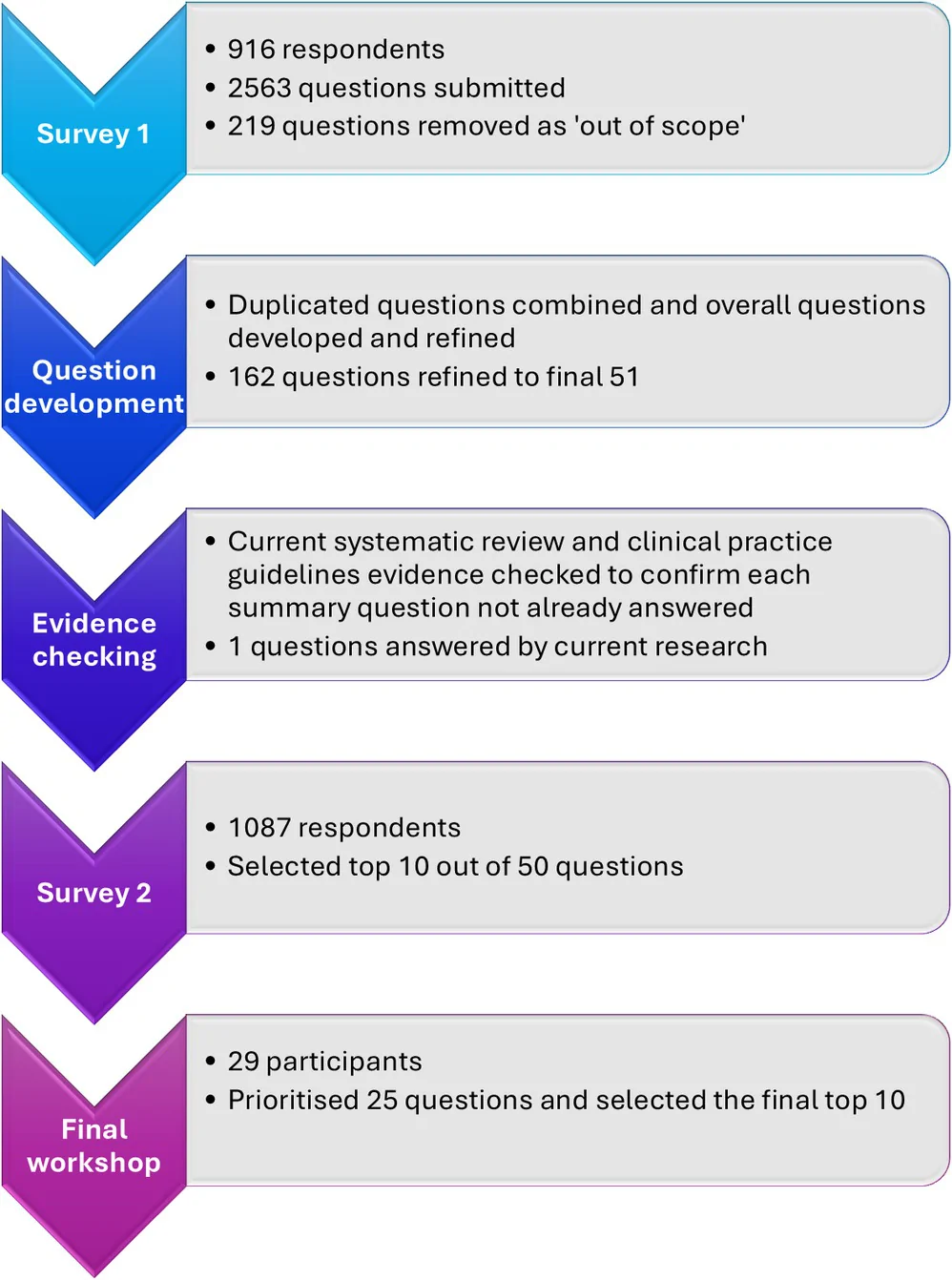
Overall process of the food allergy in children priority setting partnership.

### 2.2 Scope

The objective of the PSP was to identify unanswered research questions about food allergy in children from birth to 19 years of age. The scope was kept as broad as possible to include: all types of food allergy (immediate, delayed or mixed) in children aged 0–19 years at onset; cause, prevention, diagnosis, management, treatment, psychosocial impact and any aspect of the care pathway or configuration/access to health services. Out of scope were food intolerance and coeliac disease, food labelling, issues not relevant to the UK and access to private services [16].

### 2.3 Steering Group

A multidisciplinary steering group was established (*n* = 19), comprising two young people with food allergy, three parents of children with food allergy, eight healthcare professionals (including a paediatric allergist, GP with interest in allergy, paediatric allergy dietitian, allergy nurse specialist, consultant dermatologist, midwife, health visitor and pharmacist), representatives from four relevant charities (Allergy UK, Anaphylaxis UK, Natasha Allergy Research Foundation and Eczema Outreach Support), and the coordinating team (RMP, MJR). The steering group was chaired by an experienced JLA Advisor (JG) and was responsible for agreeing the scope of the PSP, overseeing survey design and recruitment, formulating the overall research questions, providing expert/lived experience opinion on evidence and guiding dissemination. The steering group helped identify potential partners, including professional organisations and young person networks, to ensure surveys achieved as wide a reach as possible. The steering group met regularly via Zoom to oversee the process.

### 2.4 Step 1: Identification of Research Uncertainties

An initial online survey (www.onlinesurveys.ac.uk) was developed by the steering group and piloted with patient steering group members. The following groups were invited to participate:

- Children and young people with food allergy.
- Parents and carers of children with food allergy.
- Adults with food allergy diagnosed in childhood.
- Healthcare professionals are involved in the care of children with food allergy.

Survey logic was used to present age‐appropriate wording and graphics. Children < 16 years of age were asked “Please write something that matters to you about having a food allergy” whilst adults with food allergy, parents and clinicians were asked to give specific research questions: “Please write your first question about food allergy”.

Respondents could submit up to 5 questions and basic demographic data was collected. In order to get insight from younger children, children could contribute through parental surveys and parents were encouraged to involve and consult their children if they felt it was appropriate. In addition, we held an online children's focus group with children with eczema and food allergy as part of the Eczema Outreach Supports online High Five club. It was run by an independent facilitator and observed by RMP. We asked children to describe how their food allergy had impacted them and what mattered most to them about having a food allergy. Summary notes were collated from the focus group and used to develop the overall summary questions alongside the submissions from the first survey. In the final workshop, question cards derived from the children's focus group were marked to indicate that they were “*Important in children's focus group” to ensure their perspectives were considered throughout and the final questions were child‐informed.

The survey was open for 3 months (between October 2024 and January 2025) and promoted nationally through the steering group and partner organisations (e.g., patient organisations, clinical networks, professional bodies) newsletters, emails, social media and meetings and conferences. The demographic characteristics of respondents were regularly reviewed by the steering group and guided targeted promotion to under‐represented groups, such as targeted social media adverts to fathers of children with food allergy.

### 2.5 Step 2: Development of Summary Questions

Submitted questions were collated, reviewed, and double coded into overall themes by RMP and at least one other steering group member. Questions were reviewed iteratively with overall coded themes reviewed and agreed upon by the steering group. Out‐of‐scope submissions were excluded. Similar questions were combined to form indicative summary questions while retaining the original intent and wording as closely as possible. The findings from the children's focus group were reviewed to ensure summary questions were included that addressed the issues raised. The final questions were reviewed by the steering group and three Centre for Applied Excellence in Skin and Allergy Research (CAESAR) Patient Advisory Group members to ensure they were clear and easy to understand. A number of changes were made following this feedback.

### 2.6 Step 3: Literature Review

Evidence checking was undertaken to confirm that each summary question represented a genuine uncertainty not already answered by existing systematic reviews or clinical practice guidelines. An expert in systematic reviews and evidence synthesis (FS) developed and undertook a search for relevant literature based on the keywords: food allergy (topic), children (population), and structured reviews and guidelines (publication type). On the 4, April 2025, the search was run in PROSPERO, Epistemonikos, Cochrane Database of Systematic Reviews, Google Scholar, PubMed, Trip Database, and NICE (available at the JLA website [16]), restricted to the last 5 years as per JLA guidance [15]. This identified 510 records which were screened for relevance, and 205 full text articles were selected. These were reviewed for information that addressed or provided answers to the question short list. Relevant data were extracted and copied into a Microsoft Excel spreadsheet against each question. The evidence found against each summary question was reviewed to assess if it was ‘answered’ (sufficient evidence exists to answer the question); ‘partially answered’ (evidence exists but it is incomplete/inconclusive and/or leaves important sub questions unanswered); or ‘unanswered’ (absent evidence or insufficient to answer the question). Uncertainties and future research requirements explicitly noted in the reviews/guidelines were extracted.

### 2.7 Step 4: Interim Prioritisation

The final list of 50 questions formed a second prioritisation survey (run on REDCap, https://project‐redcap.org/) with questions grouped into sections (e.g., Cause, Prevention, Diagnosis, Treatment, Anaphylaxis, Medication, Impact). The survey was open for 4 months (May 2025–August 2025). Those from survey 1 were invited to take part again and the survey was repromoted through the same channels as survey 1. Respondents were asked to choose up to 10 questions that were the most important to them. The order of the questions was varied, with half seeing questions 1–28 first and half seeing questions 29–50 first. The overall scores for each question were calculated for each stakeholder group: children/young people, adults/parents/carers, and healthcare professionals. The questions were then ranked from the highest scoring to the lowest scoring with the highest‐ranked 25 questions for each stakeholder group shortlisted for discussion at the final workshop.

### 2.8 Step 5: Final Priority Setting Workshop

A final in‐person consensus workshop facilitated by three independent JLA Advisors (including JG) was held on the 3rd of October 2025 in Birmingham, using a modified nominal group technique [17]. Participants were invited from those who had indicated that they were willing to take part in the final workshop in survey 2. Participants were selected to include a balanced mix of patients, carers, and healthcare professionals as well as a range of genders and ethnicities. RMP and MJR observed the process, taking notes on the discussions but did not contribute to the decision making.

Prior to the workshop, participants were asked to rank the importance of questions from 1 to 25 in order of importance and select their 3 most important questions. Participants were split into three pre‐allocated groups ensuring a balance of multidisciplinary professionals, young people and parents. In each group, participants shared their three highest and lowest ranking questions. Discussion followed, and each group ordered the 25 questions from highest to lowest. The ranking of the 25 questions from the three groups was combined. In the following session, in new group compositions, the combined consensus ranking was the starting point for discussion. Following this second round of discussion, the group rankings were again collated to produce the final ranking of all 25 questions highlighting the “top 10”.

## 3 Results

### 3.1 Identification of Research Uncertainties

Nine hundred and sixteen people submitted 2563 questions (Table 1). Respondents were mostly parents/carers 538 (59%) and health care professionals 182 (20%) but also included adults 74 (8%) with food allergy diagnosed in childhood, children completing the survey independently 32 (2%), children who inputted into their parent's survey 84 (9%) and those who indicated they were “someone else” 6 (1%), usually an allergy clinician and parent. A range of health care professionals took part. Respondents were largely White, 776 (85%), 60 (7%) Asian/Asian British, or 27 (3%) Mixed/multiple ethnic groups.

**TABLE 1:** Roles and demographics of survey respondents and workshop participants.

Role, *n* (%)	Survey 1 (*n* = 916)	Survey 2 (*n* = 1087)	Workshop (*n* = 29)
Child (alone)	32 (3%)	15 (1%)	0
Child (input with parent)	84 (9%)	82 (8%)	3 (10%)*
Parent/Carer	538 (59%)	509 (47%)	7 (24%)
Adult	74 (8%)	107 (10%)	4 (14%)***
Health Care Professional	182 (20%)	374 (34%)	15 (52%)
General Practitioner (GP)	44 (24%)	96 (26%)	2 (13%)
Paediatrician	40 (22%)	49 (13%)	4 (27%)**
Allergist	22 (12%)	17 (5%)	4 (27%)**
Nurse	7 (4%)	10 (3%)	0
Specialist Nurse	10 (5%)	38 (10%)	5 (33%)
Dietician	26 (14%)	37 (10%)	2 (13%)
Dermatologist	15 (8%)	10 (3%)	0
Midwife	0	0	0
Health Visitor	3 (2%)	54 (14%)	1 (7%)
Pharmacist	2 (1%)	9 (2%)	0
Emergency Doctor	2 (1%)	29 (8%)	1 (7%)
Other	9 (5%)	18 (5%)	0
Missing	2 (1%)	7 (2%)	0
Other	6 (1%)	0	0
Demographics, *n* (%)			
Male	133 (15%)	187 (17%)	7 (24%)
Female	754 (82%)	863 (79%)	22 (76%)
Non‐binary	4 (0%)	2 (0%)	0
Prefer not to say	8 (1%)	10 (1%)	0
Prefer to self describe	3 (0%)	1 (0%)	0
Missing	14 (2%)	24 (2%)	0
White	776 (85%)	887 (82%)	25 (86%)
Mixed/multiple ethnic groups	27 (3%)	48 (4%)	1 (3%)
Asian/Asian British	60 (7%)	64 (6%)	2 (7%)
Black/African/Caribbean/Black British	11 (1%)	28 (3%)	0
Other ethnic group	11 (1%)	14 (1%)	0
Prefer not to say	17 (2%)	22 (2%)	1 (3%)
Missing	14 (2%)	24 (2%)	0
Under 8 years old	21 (2%)	27 (2%)	0
8–11 years old	46 (5%)	35 (3%)	0
12–16 years old	28 (3%)	27 (2%)	1 (3%)
16–21 years old	21 (2%)	27 (2%)	3 (10%)
22–30 years old	57 (6%)	75 (7%)	3 (10%)
31–40 years old	314 (34%)	307 (28%)	4 (14%)
41–50 years old	273 (30%)	360 (33%)	13 (45%)
51–60 years old	96 (10%)	157 (14%)	5 (17%)
61–70 years old	14 (2%)	34 (3%)	0
Over 70 years old	2 (0%)	6 (1%)	0
Missing	44 (5%)	32 (32%)	0

* Attended workshop with parent.

** Four healthcare professionals were ‘paediatric allergists’ and therefore included in both the ‘Paediatrician’ and ‘Allergist’ categories.

*** Young adult (aged 21–26).

Two hundred and nineteen questions were judged to be out of scope and removed. These included questions on food labelling, travelling and managing food allergies abroad, government policies and regulations, food allergies in adults, or non‐food allergy questions.

The remaining questions were put into 13 major categories: cause, prevalence, prevention, allergens, diagnosis, treatment, anaphylaxis, medication, impact, communication, healthcare services, eating out and schools. A first review of submissions generated 162 indicative questions. Through an iterative process, reviewing the original submissions with steering group and patient and public input, the list was further consolidated into 51 final questions. One question was considered to be already answered by current research. Thirty‐one questions were considered to be partly answered, and 19 were considered to have no research evidence (final data spreadsheet available via the JLA website—see Availability of data and materials).

Seven children with food allergy aged 8–11 years (2 females, 5 males) participated in the online workshop. They talked about the social impact of food allergies and feeling left out, problems eating at school and outside of home, the emotional impact, wanting to find a “cure” and “why me?” All these topics were represented in the 50 indicative questions taken through to the second survey.

### 3.2 Interim Prioritisation

One thousand and eighty‐seven respondents took part (Table 1). Respondents were mostly parents/carers 509 (47%) and healthcare professionals 374 (34%) but also included adults 107 (10%) with a food allergy diagnosed in childhood, children completing the survey independently 15 (1%) and children who input into their parents' survey 82 (8%). A range of health care professionals took part (Table 1). Respondents were largely White, 887 (82%); 64 (6%) were Asian/Asian British, and 48 (4%) mixed/multiple ethnic groups.

Questions that were ranked highly by both patients, parents/carers and healthcare professionals were: what factors increase the risk of a child developing food allergy, the most effective treatments for food allergy, the best ways to introduce foods to build tolerance and how to prevent food allergy. Questions ranked highly by families but lower by healthcare professionals included: how eating out can be made safer, the impact of living with food allergy and support in care settings. Questions ranked highly by healthcare professionals but lower by families included: type of diet causing food allergy, the true prevalence of Cow's Milk Protein Allergy (CMPA) and the most effective way to improve the early diagnosis of food allergy.

The most important questions to patients/parents/carers and healthcare professionals resulted in 25 questions, which were taken into the final workshop; these included the issues raised in the children's focus group.

### 3.3 Final Workshop

Twenty‐nine people living with or managing food allergies participated in the final workshop (Table 1): 3 young people (aged 18 years and below), 4 young adults (21–26 years), 7 parents and 15 healthcare professionals including four consultant paediatric allergists, three allergy nurses, two dietitians, two dermatology nurses, two GPs, one health visitor and one urgent care practitioner. One additional parent was unable to attend.

Discussions during the workshop centred around ensuring the perspectives from the children's focus group and different aspects of the food allergy pathway: prevention, diagnosis, treatment and impact were all included in the final top 10. There was strong consensus across all attendees that prevention should be the number one priority. Many patients shared stories about poor early experiences in primary care and healthcare professionals agreed that better diagnosis and management in primary care is achievable. Whilst healthcare professionals ranked “safety when eating out” lower, lay members prioritised this because they are “living with this now” and its impact on their quality of life. Discussions around questions relating to improving access to allergy teams led to a lower ranking as participants felt they related to the NHS structure rather than research. Workshop participants felt “How does living with a food allergy impact children, young people and their families?” should be included in the top 10 as it captured how the quality of life of families with food allergy can be improved as well as understanding its impact. It was felt that the scope for improving awareness (“How can food allergy awareness and education be improved in healthcare professionals and parents?”) could be extended from healthcare professionals and parents to all members of the public.

### 3.4 Top 10 and 25 Research Priorities

The final agreed top 10 research priorities for childhood food allergy are listed in Table 2. These priorities span the full patient journey including prevention, diagnosis, treatment, impact on quality of life and eating out as well as safety and inclusion in childcare/school settings. Table 3 includes the additional 15 questions ranked in the final workshop. A video (FoodAllergiesVideo‐PALSTOWE3) of the full PSP process can be found at: https://foodallergyinchildrenpsp.blogs.bristol.ac.uk/ (Figure 2 and Video 1). Further details and example original submissions are included in Data S1.

**TABLE 2:** Top 10 research priorities for childhood food allergy.

What are the safest, most effective and cost‐effective ways to prevent food allergy in children? What is the most effective way to improve the early diagnosis and care of children with food allergy in primary care and the community? (e.g., GP, health visitor, dietitian support and advice beyond total food allergen avoidance) What are the safest, most effective and cost‐effective treatments for children with known food allergy? For example, immunotherapy, food ladders, medication What factors increase the risk of a child developing food allergy and influence whether food allergy gets worse or is outgrown? How can eating out in restaurants and cafes be made safer and more inclusive for families with food allergies? Including: training, awareness, alternatives, allergy menus and allergy rating? What is the most effective way to ensure that nurseries and schools support children with food allergy and their families? Including: staff training, emergency medication access, inclusive meal planning and allergen free activities. How does living with a food allergy impact children, young people and their families? Including: eating disorders; nutrition, growth & development; quality of life; social and emotional wellbeing and the challenges of eating out. How can emergency treatment for anaphylaxis be improved? For example, through public awareness of, access to and use of adrenaline/epinephrine How can food allergy awareness and education be improved in healthcare professionals and parents? What are the best ways to introduce food allergens to desensitise/build tolerance to treat children with food allergy? Including: age, dose and frequency.

**TABLE 3:** Priorities ranked 11 to 25 for childhood food allergy.

11 How can the long‐term care and follow up of children with food allergy be improved through childhood and the transition to adulthood? 12 How can we better distinguish between food allergy, food intolerance and sensitisation to foods? [Explanatory note: Food allergies involve the immune system and food intolerances don't. Sensitisation is when the immune system has produced antibodies to a food, but the person does not have symptoms when they eat it.] 13 What is the relationship between food allergy and other conditions and how do they affect each other? For example, eczema, asthma, colic, mast cell diseases and neurodiversity such as autism. 14 What factors increase the risk of a severe allergic reaction (anaphylaxis)? 15 How can equal access to allergy teams, diagnosis and treatments such as oral immunotherapy be achieved? 16 How do things like a mother's diet, how a baby is born, vaccines or early use of antibiotics or formula milk affect whether a child gets food allergies? 17 Can children at higher risk of food allergies (e.g., family history, eczema, existing food allergy) be identified early and their risk reduced? 18 How effective are antihistamines, do they work for everyone, prevent severe reactions to food and can they be used daily without developing a tolerance? 19 What are the most effective treatments for anaphylaxis, including anaphylaxis that persists after initial treatment with adrenaline/epinephrine? 20 What are the long‐term benefits and risks of a child consuming a food they are allergic to through food ladders, immunotherapy, or accidental exposure? 21 How does a child's diet affect whether they get food allergy? In particular, how early food allergens are given and how often. 22 Do babies react to allergens in mother's breast milk? 23 How common is Cow's Milk Protein Allergy (CMPA) in breast fed, formula fed and mix fed babies? 24 How can communication about a child's food allergy be improved between parents and different care settings? For example, extended family, nurseries and schools? 25 Has the number of children with different types of food allergy changed over time and if so why? For example, immediate (IgE), delayed (non‐IgE), single or multiple foods.

**FIGURE 2:**
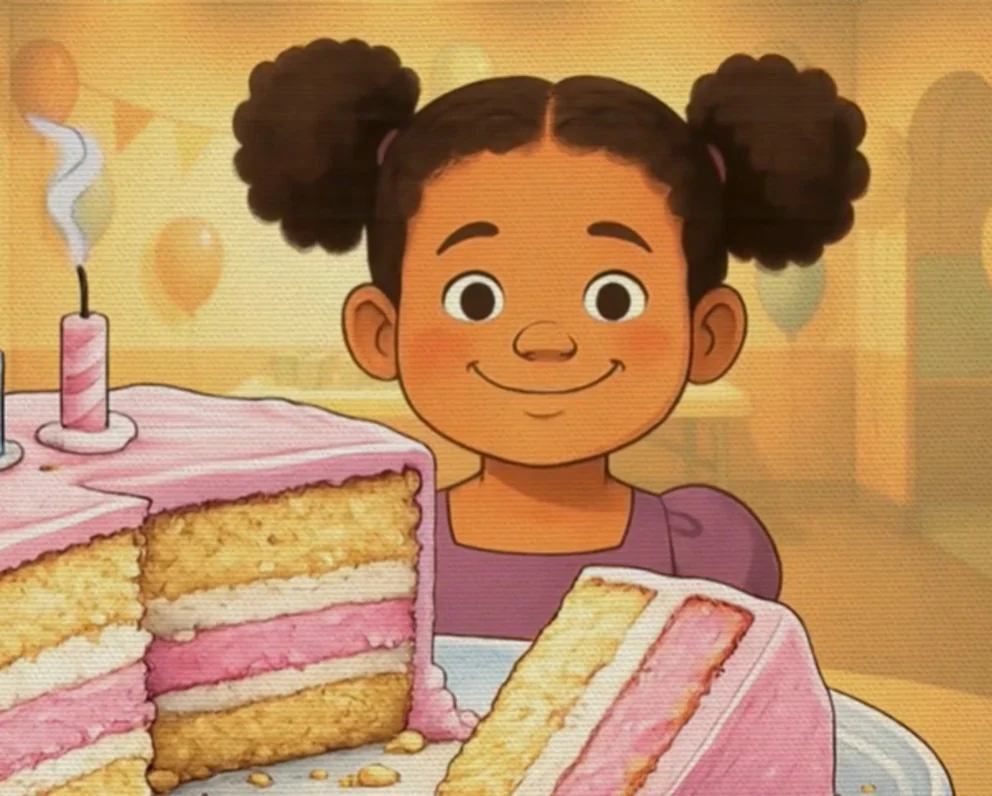
Video‐Top 10 research priorities for childhood food allergy.

**VIDEO 1:** Behind every research question is a child, a family, and a story. This project brought together children and young people, parents and carers, healthcare professionals, researchers, educators, and support organisations to identify the most important unanswered questions in child food allergy research. Through a nationwide survey, we gathered 2563 questions and uncertainties from people with lived experience and those involved in care. These were carefully reviewed, refined and prioritised through a collaborative process, culminating in a final consensus workshop where patients, families and professionals worked together to agree on the Top 10 research priorities. By listening to those most affected by food allergy, we can ensure future research focuses on what matters most: preventing food allergies, improving diagnosis and treatment, making schools and public spaces safer, and helping children and families live with greater confidence and less fear. #FoodAllergy #ResearchPriorities #PatientVoice #ChildHealth #FoodAllergyResearch #PPIE #HealthResearch #FoodAllergyAwareness. Video content can be viewed at https://onlinelibrary.wiley.com/doi/10.1111/cea.70377.

## 4 Discussion

This represents the first nationally coordinated exercise to define shared research priorities for childhood food allergy using a transparent, stakeholder‐driven methodology. The resulting top 10 priorities highlight areas where current evidence is insufficient and where future research is most likely to deliver meaningful benefit to children with food allergy, their families, and healthcare professionals supporting their care.

A key finding is the prominence of priorities related not only to prevention and treatment interventions, but also to supporting early diagnosis and care in the community, psychosocial wellbeing, and practical management in everyday settings including nursery, school, and eating out. This highlights the multifaceted burden of childhood food allergy and the need for research that explores lived experience and public health interventions to improve awareness and safety in public settings.

Priorities 1 (prevention), 3 (treatment), and 10 (optimal timing/dose of food allergens) align with the key future research recommendations from a recent systematic review of ways to prevent food allergy including: the need to validate findings on the benefits of early introduction of peanuts/eggs in different ethnic groups and investigate the mechanisms that may modulate immune development in food allergy such as gut health [18]. Three recent systematic reviews on the burden and impact of food allergy [7, 8, 19] include key future research suggestions on the need for longitudinal studies tracking nutrient deficits, growth and psychosocial trajectories and predictors of distress, how these may differ between genders, age groups and cultural contexts and interventions to improve nutrition, reduce anxiety, improve coping, and support families. This aligns with research priority 7, the need to understand the impact of having a food allergy. Finally, recent systematic reviews have additionally called for the need to involve and include children in future research to improve food allergy literacy in children and safety management in schools, supporting priority 6 (safety in care settings) [20, 21].

### 4.1 Strengths and Limitations

Setting up a diverse steering group was a key strength of this PSP as it enabled us to promote the surveys through the UK's largest allergy and eczema charities as well as other patient and professional organisations. We had high stakeholder engagement (916 in survey 1 and 1087 in survey 2) which is higher than recent comparable UK PSPs. We adhered to established JLA methodology throughout the process and had independent facilitation from a JLA advisor.

The equal weighting of patient, carer, and clinician perspectives strengthens the relevance and legitimacy of the outcome. The top research questions identified in this study give a voice to those directly impacted by food allergy to ensure future research focuses on the questions that matter most. Establishing the order of priority for research questions provides a clear guide for research leaders and funders to allocate resources.

In keeping with JLA PSP methodology, we did not attempt to verify survey respondents' identity or, in the case of patients/carers, their diagnosis. We made it clear on the first survey that we wanted to hear from people with experience of childhood onset food allergy, and not food intolerances, food sensitivity or celiac disease. However, we still invited questions about distinguishing these conditions or if patients/carers were “unsure if they had a food allergy or something else.” Although largely in line with UK ethnicity data [22], limitations include potential under‐representation of certain groups, such as very young children, males and individuals from minority ethnic communities. This limitation is similar to many other PSPs that have reported a low proportion of responses from males and people from a range of ethnicities [12, 23, 24]. Most respondents were white and female even following efforts to increase participation from males and fathers through social media advertising. Engaging those from ethnic minority backgrounds through national networks such as the Caribbean and African Health Network and more locally, funding the time of ambassadors in Caafi Health (a Bristol‐based community interest company) facilitated a slight increase in the number of minority ethnic respondents. Future work should include methods to engage these groups further, targeting local community organisations throughout the UK.

A focus group was held to understand the experiences and views of children, and allowing child input into parental surveys was key to gaining further child engagement. However, fewer children took part independently in the surveys. Future PSPs involving children should consider child‐friendly data collection methods; focus groups may be more successful, as in our experience few children participated in surveys independently.

The initial scope of the PSP was kept broad to allow for questions covering the whole pathway and patient experience; however, this resulted in a wide range of questions and a substantial workload to sort and combine them. The final top 25 questions are broad to encompass a range of original submissions from survey 1 to reduce the burden for participants in survey 2 to only 50 questions. Therefore, further refinement and development of more specific research questions is required to take forward as feasible research projects. There is potential for confirmation bias as stakeholders might have been exposed to existing dominant topics in food allergy (e.g., early allergen introduction, access to specialist care). The James Lind Alliance methodology mitigates this risk through open‐ended survey design to avoid priming, inductive analysis, and inclusion of diverse stakeholder groups. However, we recognise that some final priorities may reflect the consolidation of existing concerns and topics rather than entirely novel uncertainties, but these remain important to stakeholders and are currently insufficiently addressed.

## 5 Conclusions

The priorities identified through this PSP provide a clear roadmap for researchers, funders, and policymakers. Alignment of future funding calls with these priorities will ensure that research investments address questions that matter most to those directly affected.

## Author Contributions

Roxanne M. Parslow: Responsible for the day‐to‐day running of the PSP. Designing and administering the surveys, focus group and workshop, led on analysing the survey responses and categorising them into in‐out of scope, indicative questions and calculating rankings. Responsible for writing the first draft and revising the manuscript. Farhad Shokraneh: Led on a rigorous search of the existing research evidence. Reviewed the manuscript Sara Brown: Provided overall guidance for the project as a steering group member contributing to the promotion of the surveys, decisions on the scope of the PSP, formation of indicative questions, and literature review. Reviewed the manuscript. Mary Feeney: Provided overall guidance for the project as a steering group member contributing to promotion of the surveys, decisions on the scope of the PSP, formation of indicative questions and literature review. Reviewed the manuscript. Graham Roberts: Provided overall guidance for the project as a steering group member contributing to the promotion of the surveys, decisions on the scope of the PSP, formation of indicative questions, and literature review. Reviewed the manuscript. Hora Soltani: Provided overall guidance for the project as a steering group member contributing to the promotion of the surveys, decisions on the scope of the PSP, formation of indicative questions and literature review. Reviewed the manuscript. Joanne Walsh: Provided overall guidance for the project as a steering group member contributing to the promotion of the surveys, decisions on the scope of the PSP, formation of indicative questions and literature review. Reviewed the manuscript. Amy Dobson: Provided overall guidance for the project as a steering group member contributing to the promotion of the surveys, decisions on the scope of the PSP, formation of indicative questions, and literature review. Reviewed the manuscript. Emma Vaughan: Provided overall guidance for the project as a steering group member contributing to the promotion of the surveys, decisions on the scope of the PSP, formation of indicative questions, and literature review. Reviewed the manuscript. Deborah Marriage: Provided overall guidance for the project as a steering group member contributing to the promotion of the surveys, decisions on the scope of the PSP, formation of indicative questions and literature review. Reviewed the manuscript. Suzi Holland: Provided overall guidance for the project as a steering group member contributing to the promotion of the surveys, decisions on the scope of the PSP, formation of indicative questions and literature review. Reviewed the manuscript. Mary Kelly: Provided overall guidance for the project as a steering group member contributing to the promotion of the surveys, decisions on the scope of the PSP, formation of indicative questions and literature review. Reviewed the manuscript. Holly Shaw: Provided overall guidance for the project as a steering group member contributing to the promotion of the surveys, decisions on the scope of the PSP, formation of indicative questions and literature review. Reviewed the manuscript. Alicia Kennedy: Provided overall guidance for the project as a steering group member contributing to promotion of the surveys, decisions on the scope of the PSP, formation of indicative questions and literature review. Reviewed the manuscript. Eden Byrne: Provided overall guidance for the project as a steering group member contributing to the promotion of the surveys, decisions on the scope of the PSP, formation of indicative questions and literature review. Reviewed the manuscript. Liliana Jackson: Provided overall guidance for the project as a steering group member contributing to the promotion of the surveys, decisions on the scope of the PSP, formation of indicative questions and literature review. Reviewed the manuscript. Gail Jackson: Provided overall guidance for the project as a steering group member contributing to the promotion of the surveys, decisions on the scope of the PSP, formation of indicative questions and literature review. Reviewed the manuscript. Lauren Gluyas: Provided overall guidance for the project as a steering group member contributing to the promotion of the surveys, decisions on the scope of the PSP, formation of indicative questions, and literature review. Reviewed the manuscript. Jonathan Gower: Provided overall guidance for the project as the James Lind Alliance advisor. Reviewed the manuscript. Alyson Huntley: Led on a rigorous search of the existing research evidence. Reviewed the manuscript. Matthew J. Ridd: Secured the initial funding for the PSP, provided oversight and guidance on the project, checked the systematic review evidence against the overall PSP research questions. Reviewed the manuscript.

## Funding

This project was funded by an NIHR Research Professorship (NIHR303123). The views expressed are those of the author(s) and not necessarily those of the NIHR or the Department of Health and Social Care.

## Ethics Statement

Ethical approval was obtained from the University of Bristol Health Sciences Research Ethics Committee (FREC) (Ref: 21773).

## Conflicts of Interest

G.R. was the President of BSACI. J.W. is involved in the task force for feeding difficulties in children with allergy. S.J.B. holds a Wellcome Trust Senior Research Fellowship (220,875/Z/20/Z).

## Supporting information


**Data S1:** Further detail and example original submissions of the top 10 research priorities in childhood food allergy.

## Data Availability

A spreadsheet which includes the overall question, some examples of the original submissions and examples and references of identified existing evidence is freely available via the James Lind Alliance Website: https://www.jla.nihr.ac.uk/priority‐setting‐partnerships/food‐allergy‐children#tab‐key‐documents.
