# Supplementary material for: Research Priorities for Children With Food Allergy: A UK James Lind Alliance Priority Setting Partnership
Source: Clin Exp Allergy. 2026 Jul 5;56(11):1401–10. doi: 10.1111/cea.70377 (PMC13727359; doi:10.1111/cea.70377)
Supplement: Supplementary file 1 [file CEA-56-1401-s001.docx]

## Supplementary 1: Further detail and example original submissions of the top 10 research priorities in childhood food allergy

1. **What are the safest, most effective and cost-effective ways to prevent food allergy in children?**

Survey 1 respondents wanted to know if there were things that could be done in pregnancy, while breastfeeding and in early infancy that could prevent food allergy from developing. Supporting mothers to continue breastfeeding was important. Submissions also included questions about preventative measures in high-risk infants (those with a family history and with eczema). Interventions that are cost effective was also raised as important.

Original submissions:

“Could breastfeeding be a way to introduce the proteins in a gentler way to my daughter’s body because they are partially broken down/filtered through me/my milk?” (Parent)

“How to reduce the likelihood of a child having a food allergy (especially a child who is high risk due to allergies in family) through pregnancy and early life e.g. nutrition, supplements taken in pregnancy” (Parent)

“How can we better prevent allergy e.g. faster access to allergy specialists, testing, prevention advice” (HCP)

“What are the most cost-effective interventions to prevent allergies?” (HCP)

2. **What is the most effective way to improve the early diagnosis and care of children with food allergy in primary care and the community? (e.g. GP, health visitor, dietitian support and advice beyond total food allergen avoidance)**

A need for improved early diagnosis and care of infants with food allergy infants was clear from survey 1. Submissions referenced long waiting lists to see allergy specialists and parents wanted to be taken seriously by doctors and avoid life-threatening reactions. Clinicians wanted testing to establish if symptoms in babies were allergy driven. Advice beyond simply avoiding suspected food allergens was felt to be particularly important and survey respondents wanted to know if this could be carried out in primary care by GPs or the community by health visitors and dietitians.

Original submissions:

“How to make a clear diagnosis of a child with food allergy? developing better diagnostic tools and protocols for identifying food allergies in children at an early stage” (HCP)

“My second question is regarding how difficult it is to get a diagnosis and also to be taken seriously by the medical profession when one raises the possibility of one’s baby / child having an allergy.” (Parent)

“How can community based healthcare professionals be up skilled in a meaningful way to support patients with food allergies over the course of their practice?” (HCP)

“Managing food allergy proactively rather than just using avoidance as a strategy” (Parent)

3. **What are the safest, most effective and cost-effective treatments for children with known food allergy? For example, immunotherapy, food ladders, medication**

Developing treatments that not only increase the tolerance of allergic foods to help manage accidental exposure, but a permanent cure was felt to be important. There was a need to understand the safety and long-term impacts of treatments such as oral immunotherapy on child and family quality of life. Survey respondents wanted to know what treatments may be most effective for different age groups. They wanted to further research into treatments that may prevent severe reactions. It was felt that there is a need for further research into specific treatments such as immunotherapy, food ladders, medication, immunosuppressants, mRNA, sublingual immunotherapy and gene therapy. Low-cost treatment methods affordable to the NHS was important.

Original submissions:

“Can you find a cure.” (Child)

“How can we develop immunotherapy or other therapy to all his allergens, which is a definite permanent cure?” (Parent)

“What is the best treatment for allergy at different ages e.g OIT, palforzia etc” (Parent)

“long term impact of OIT for child and family QOL” (HCP)

“What is the optimum way of doing milk, egg and wheat immunotherapy, in terms of safety and practicality” (HCP)

4. **What factors increase the risk of a child developing food allergy and influence whether food allergy gets worse or is outgrown?**

The cause of food allergies and “Why do some people get allergies and others do not?” was a key question posed in survey 1. There is a need to understand the risk factors for developing food allergy beyond simply having a family history and survey responses included questions around: gender, ethnicity, socio-economic status, immunity, infections and surgery. Additionally, environmental factors to explain why food allergies are more common in some countries and settings e.g. cultural beliefs, family diet and migration. Further research is needed on the likelihood and timelines for outgrowing food allergies, particularly in those that have anaphylaxis, multiple allergies, other conditions such as eczema and asthma or different types of allergies (IgE or non-IgE) and different types of foods. Finally, the likelihood of food allergies getting worse and why was also a clear theme from survey 1.

Original submissions:

“How did my son acquire a nut allergy when his five siblings did not? Are there any lifestyle factors that caused his allergy that could have been prevented? (Parent)

“What are the most common triggers for food allergies in children, and how do they vary by age, region, or ethnicity?” (HCP)

“If someone has multiple allergies are they less likely to outgrow them?” (Parent)

“Parents want to know about outgrowing food allergy… I find the data and guidelines conflicting and confusing. What should we be telling parents and young people with food allergies?” (HCP)

“What causes someone who has food allergies to get worse with foods rather than better? For example my son was allergic to two foods and over the years he has become allergic to more food.” (Parent)

5. **How can eating out in restaurants and cafes be made safer and more inclusive for families with food allergies? Including: training, awareness, alternatives, allergy menus and allergy rating?**

Many survey responses included negative experiences of eating out with restaurants not being ‘allergy friendly’, staff not taking food allergies seriously, not having the correct knowledge and a result, families not feeling safe enough to eat out. Future research should focus on how eating out could be made safer and easier for people with food allergies. How can menus be made more inclusive to offer alternatives for those with allergies? What changes are required to equipment and food preparation to reduce the chance of cross contamination. How can food menus be improved to be clearer for those with food allergies (allergy symbols, menus, listing ingredients and ratings). Can training be provided to staff serving food to improve their awareness and understanding of food allergies?

Original submissions:

“How do I eat safely when I'm away from home on school trips or when I'm not with my parents?” (Child)

“Can we introduce a national (and eventually, global) awareness and movement regarding food allergies, and ensure that strict regulations are in place in restaurant kitchens and cafes” (Adult)

“Should all businesses/organisations/staff that provide food have allergy training and EpiPen training? I find some organisations/venues/staff don't take allergies seriously and act like we are being unreasonable when asking for food/drink that is safe for my child. Some organisations have even turned us away due to my child's allergies which upsets her.” (Parent)

“Would partnerships with restaurants and food retails help advocate a more suitable allergy menu and understanding in order to support allergies and diminish the views that people with allergies are just "doing it for fashion"?” (Parent).

6. **What is the most effective way to ensure that nurseries and schools support children with food allergy and their families? Including: staff training, emergency medication access, inclusive meal planning and allergen free activities.**

It is important not only to keep children with food allergies safe in care settings but included. How to improve awareness in schools so children can be provided with food alternatives, can access free school meals and be included in school activities involving food were key questions from survey 1. How can communication between healthcare and care settings be improved to increase understanding of medication recommendations such as carrying two epinephrine/ adrenaline devices, easy access to medication and what to do in the event of a reaction? What training needs to be provided to school staff to ensure they understand food allergy, how to keep children safe and what to do in the event of a reaction?

Original submissions:

“At school lunch all the allergies are mixed together, I like cheese but cheese is dairy so I can't have it so everyday I have to have a packed lunch while I watch the other children eating yummy cheese paninis” (Child)

“More awareness in schools over the feelings of children with allergies and including them instead of always being told you can’t have this. There is an alternative to everything if we care enough to look.” (Parent)

“How can we further highlight the importance to schools and GP surgeries that what school nurses recommend is from hospital recommendations such as; 2 AAIs [adrenaline] in school, care plan, other prescribed medications” (HCP)

“My daughter's primary school felt it was best to keep her EpiPens and citirazine locked away in different places and not carried with her as they had to make sure it was safe for everyone else. It was only when I got a letter from the GP, having provided information from anaphylaxis UK and Allergy UK websites and her allergy nurse before they allowed her to have it together and more easily accessible.” (Parent)

7. **How does living with a food allergy impact children, young people and their families? Including: eating disorders; nutrition, growth & development; quality of life; social and emotional wellbeing and the challenges of eating out.**

Those living with food allergies and caring for those with food allergies felt it had a wide-ranging impact not only the child/ young person but also their families. These included the impact on day-to-day quality of life and eating habits, children’s relationship with food such as sensory problems, anxiety and eating disorders. The impact of restricted diets and alternatives on nutrition, and overall physical health and development are important to understand. Survey respondents felt it is important to understand the social impacts of food allergies; feeling left out, isolated, being treated differently and bullying. Parents wanted to understand how food allergy plays a part during life transitions to independence and how to help children and young people advocate for themselves. The psychological impact of food allergy was of particular importance including the effect of having anaphylaxis, anxiety and stress on both the young person with food allergy and their parents/ carers. Further research is needed to understand the impact on different areas of life in order to understand how to help, intervene and improve the negative impacts of living with food allergy.

Original submissions:

“Impact of food allergies on selective eating and poor relationships with food.” (HCP)

“What is the long term impact of the allergy where it restricts the diet. If vitamins or supplements are used to combat any impact, what is the impact of long term reliance on these?” (Parent)

“Understanding the impact that living with a food allergy has either on the child’s life and or the parent/carer caring for them. Ranging from missed social events such as birthdays or missing out at other celebrations such as Easter, before mentioning holidays that are difficult especially with foreign languages” (Parent)

“What is the impact of food allergy on mental health. Anaphylaxis is a traumatic event - how can those experiencing those be cared for and regain trust in food.” (Adult)

8. **How can emergency treatment for anaphylaxis be improved? For example, through public awareness of, access to and use of adrenaline/ epinephrine**

A number of problems in the emergency treatment for anaphylaxis emerged from survey 1 including: differences in who is prescribed adrenaline/ epinephrine and lack of access to it in public places and lack of clear understanding and guidance on when to use adrenaline. There is also felt to be a lack of awareness about the signs and symptoms of anaphylaxis and how to treat it. There is a need to understand allergic reactions better and when a family can manage a reaction at home or when they need to go hospital. Further research into the guidance for prescribing adrenaline/ epinephrine was felt to be important. Additionally, research into ways to raise public awareness of food allergies and how to spot and treat anaphylaxis was a key priority area identified. Respondents wanted further research on treatment when adrenaline/ epinephrine does not work. Questions posed also included how poor emergency response for anaphylaxis might impact families introducing allergic foods at home.

Original submissions:

“What to do in emergency if the epipen doesn’t work? What to expect from emergency services as well if epipen doesn’t work?” (Parent and Child)

“Public and community awareness of allergy and its potential impact if not managed correctly. What is the public/community awareness regarding the signs and symptoms of anaphylaxis? Do they know how to spot it and what action to take should they see someone experiencing anaphylaxis?” (HCP)

“Uncertainty about needing an epi pen. Living rurally with no epi pen” (Child)

“Why are epi pens not routinely placed alongside defibrillators?” (Parent and child)

“Training people around me how to use an epipen. Alot of people get scared when I hear I carry an epipen” (Child)

9. **How can food allergy awareness and education be improved in healthcare professionals and parents?**

Further research is needed into how to improve food allergy education for health processionals in primary care and the community. Families felt they were often dismissed and given unhelpful advice that their allergic child “will grow out of it”. There was also felt to be a problem with healthcare professionals lacking up to date food allergy knowledge and giving outdated advice. There was also a desire to increase food allergy awareness and education among parents, particularly the interaction between breastfeeding and food allergy and the importance of introducing allergic foods early when starting solid foods. Future research should explore which settings this would be optimal, such as antenatal or postnatal classes.

Original submissions:

“How we can educate healthcare professionals to reduce being dismissed / not believed when presenting our concerns around allergy?” (Parent)

“What is the best way to reduce misleading advice healthcare professionals (eg from Midwifes, HV, GP's, Paediatric doctors etc), eg unnecessary maternal food exclusions, removal of food that is already part of the diet etc.” (HCP)

“Is there enough knowledge or education given to new parents about why introducing allergens early in weaning is important?” (HCP)

“Clearer guidelines on weaning” (HCP)

10. **What are the best ways to introduce food allergens to desensitise/ build tolerance to treat children with food allergy? Including: age, dose and frequency.**

The optimal ways to introduce and reintroduce foods through food ladders, oral immunotherapy and food challenges to treat food allergy was a key priority area identified in survey 1. Survey respondents wanted further research into the impact of low doses of allergens on reactions and helping children to outgrow food allergy. Additionally, further research into food ladders and which ones work the best, which IgE/ non IgE patients they are most suited to and protocols for food ladders at home. Respondents wanted to see further research into food challenges: when it is safe to undertake food challenges; the optimal age of the child and dose of food allergen and the feasibility of carrying out a range food challenges in hospital settings. Treatment at home also needs to be explored to avoid multiple up dosing visits in hospitals. The best way to manage multiple food allergies, in which order should food be introduced and when are food ladders or oral immunotherapy best?

Original submissions:

*“Does micro-dosing/exposing to allergens build up a tolerance?” (Parent and child)*

*“There are lots of different re-introduction ladders to use – with small differences – like missing steps, different doses – but which is *right*? It’s confusing as a patient – either the differences don’t matter (in which case say that on the ladder) or they do matter and there’s a right answer.” (Parent)*

*“What is the optimal age of introduction of different allergens in different at-risk groups and assessing the optimal dosing schedule for introduction in terms of quantity of food and frequency/duration of exposure?” (HCP)*

*“What is the most effective way of treating food allergies in young children without needing multiple up-dosing visits to hospital?” (HCP)*

*“Treatment to desensitise children/adults with allergies enough that any accidental consumption is not life threatening. More information and data on how well this works in the longer term” (Parent)*
